# mRNA vaccination targeting *AML1::ETO* fusion gene eliminates leukemia cells via activating T cells

**DOI:** 10.1038/s41375-026-02940-3

**Published:** 2026-04-10

**Authors:** Changli Zhou, Sicheng Bian, Jiuxia Pang, Huiqin Bian, Tao Cheng, Hiroaki Koyama, Bin Liu, Bing Li, William Tse, Shujun Liu

**Affiliations:** 1 Department of Medicine, The MetroHealth System, Case Western Reserve University, 2500 Metro Health Drive, Cleveland, OH 44109 USA; 2 Hormel Institute, University of Minnesota, 801 16th Ave NE, Austin, MN 55912 USA; 3 Department of Pathology, The University of Iowa, 431 Newton Road, Iowa City, IA 52242 USA; 4 Gene and Cell Therapy Institute, The MetroHealth System, Case Western Reserve University, 2500 Metro Health Drive, Cleveland, OH 44109 USA

**Keywords:** Cytokines, Haematological cancer

## Abstract

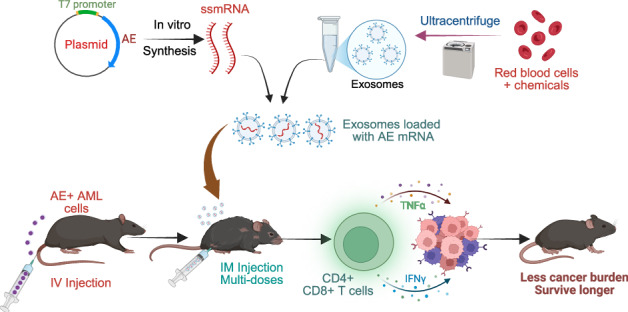

## To the Editor

The t(8;21) translocation in AML generates the non-druggable RUNX1::RUNX1T1 (AML1::ETO; AE) fusion protein. Despite initial remission, relapse rates reach 70%, especially in older patients [1]. Current immunotherapies (alloHSCT, CAR-T) are limited by long manufacturing times, toxicity [2, 3] and lacking AML-specific antigens [4]. While AE and FLT3-ITD mutations are immunogenic [5, 6], successful targeted immunotherapies remain unexplored.

mRNA vaccines offer a rapid, cost-effective alternative [7, 8]. Drawing on the success of mRNA technology in COVID-19 and recent cancer trials [9], we hypothesized that nucleoside-modified mRNA (*m*^1^Ψ mRNA) targeting fusion proteins could induce systemic anti-tumor immunity. Thus, we developed EV-AE, a nonimmunogenic *m*^1^Ψ AE mRNA-exosome complex. Intramuscular administration in an immunocompetent AE + AML murine model demonstrates its potential as a robust therapeutic solution for AE + AML.

To synthesize mRNA in vitro, we cloned AE9a or GFP gene into T7 promoter plasmids (pGEM4z-GFP-64A) containing 3’ and 5’ UTRs, then linearized via SpeI-HF digestion (Fig. S1A, B). During transcription, all uridine residues were replaced with pseudouridine (Ψ) to reduce immunogenicity [10] and enhance stability. We added a CAP2 (3´-O-Me-m7G(5’)ppp(5’)G) structure to the 5’ end to improve translatability [11] and purified the product to remove pro-inflammatory dsRNA contaminants (Fig. S1C) to decrease inflammation [12, 13]. Although purification resulted in 50% mRNA loss, high quality and integrity were maintained.

**Fig. 1:**
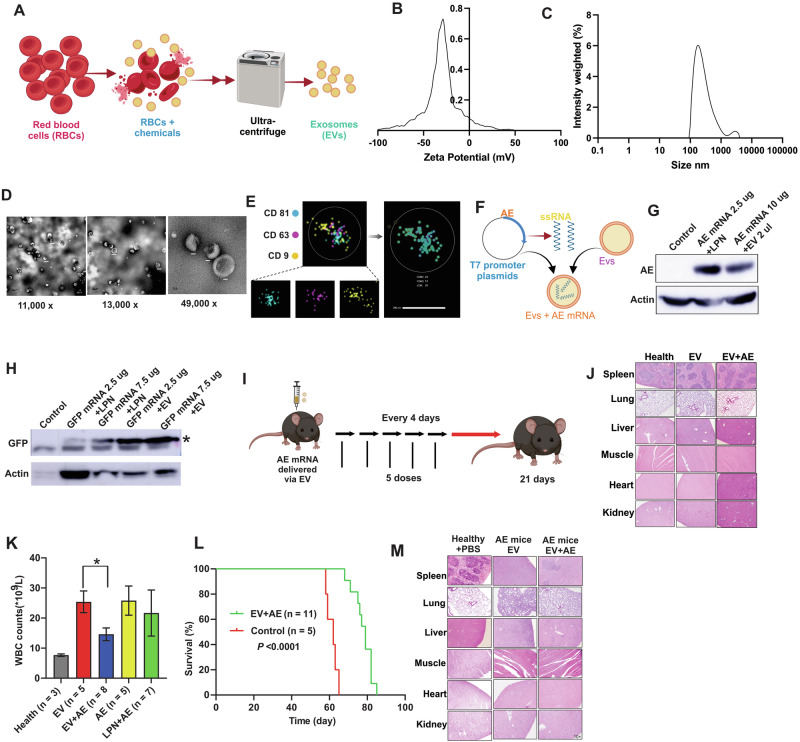
In vitro and in vivo translation assays of EVs-encapsulated mRNA. **A** Experimental design. Schematic illustrating the purification of exosomes from red blood cells. Charge (**B**) and size (**C**) distribution of EVs determined by a Nanosight nanoparticle analyzer. **D** Morphological characterization of EVs by TEM. Representative TEM images are shown at magnifications of 11,000×, 13,000×, and 49,000×. Scale bar: 200 nm. **E** Representative dSTORM images of pan-tetraspanin markers (CD9, CD63, and CD81). The number of molecules (23, 12, and 29) for each marker (CD81, CD63, and CD9) on individual EVs are indicated in the right panel. Scale bar: 200 nm. **F** Experimental design. Schematic illustrating the in vitro synthesis and uploading of mRNA to the exosomes. **G** Western blot analysis of AE protein expression in 293 T cells. Cells were transfected with AE expression DNA plasmids via LPNs or with AE expression mRNA delivered by EVs. The data shown are representative of three independent experiments. **H** Western blot analysis of GFP expression (probed with GFP antibody) in mouse muscle. Healthy C57BL/6 J mice (*n* = 3 mice/group) were primed by an IM injection of EV (control), LPN-GFP or EV-GFP mRNA. Muscle tissues were harvested 48 h post-injection. “*” indicates GFP protein bands. **I** Experimental design. Schematic illustrating the vaccination and tissue collection schedule. **J** Representative images of H&E-stained sections of spleen, lung, liver, muscle, heart, and kidney. Tissues were collected from healthy mice (*n* = 3 mice/group) vaccinated with EV or EV-mRNA. Healthy controls are the same age of mice receiving PBS injection. **K** Bar-charts showing the total WBC counts in leukemic mice four weeks after receiving treatment with EV, LPN or delivered mRNA. Healthy, untreated mice were used as controls. **L** Survival analysis in leukemic mice. Kaplan-Meier survival curves showing a significantly increased survival rate in leukemic mice treated with EV-delivered mRNA. The comparison was performed using the log-rank test. **M** Representative H&E stained sections of the spleen, lung, liver, muscle, heart, and kidney demonstrating tissue damage in vaccinated vs control mice (*n* = 3 mice/group). *P < 0.05; ns no statistically significant, dSTORM direct stochastic optical reconstruction microscopy, TEM transmission electron microscopy, WBC white blood cell counts, EV exosomes, LPN lipofectamine nanoparticles, AE AE mRNA in PBS, EV + AE AE mRNA delivered by EV, LPN-AE AE mRNA delivered by LPN, IM Intramuscular, H&E hematoxylin and eosin.

To avoid the cellular toxicity and low human delivery efficiency (1–3%) associated with intramuscular LNPs [14], we utilized exosomes (EVs) isolated from red blood cells via sequential centrifugation (Fig. 1A). Characterization confirmed a homogeneous population (80–200 nm; peak 200 nm) with a zeta potential of –20 to –30 mV (Fig. 1B, C). TME revealed typical exosome- and microvesicle-like morphologies (50–200 nm) (Fig. 1D), while Nanoimager analysis confirmed the presence of canonical exosome markers CD9, CD63, and CD81 (Fig. 1E).

To load AE9a or GFP mRNAs into EVs, we used the Exo-Fect™ Exosome Transfection Reagent. Initial supernatant analysis via agarose gel suggested the kit buffer precipitated all mRNA (Fig. S1D), subsequent RNase treatment confirmed this was not EV-related loss. Efficient loading was verified by releasing mRNA via detergent lysis of the EVs (Fig. S1E), confirming efficient mRNA loading into EVs.

To assess translational capacity, we first delivered a TX-Red positive control to 293 T cells, finding that 2 µl (2 × 10^10^ total EVs) of EVs yielded peak efficacy; higher concentrations reduced expression (Fig. S1F). mRNA delivered via EVs (Fig. 1F) resulted in efficient protein translation within 8 h, peaking at 24 h. Neither dsRNA removal nor Ψ replacement affected protein production (Fig. S1G–I). Interestingly, while in vitro translation was higher with LNP delivery than EVs (Figs. 1G and  S1J, K), in vivo intramuscular injection in C57BL/6 mice revealed that EV delivery produced higher GFP protein levels than LPNs after 48 h (Fig. 1H). These findings collectively indicate that EVs effectively deliver and translate mRNA into protein in both in vitro and in vivo contexts.

**Fig. 2:**
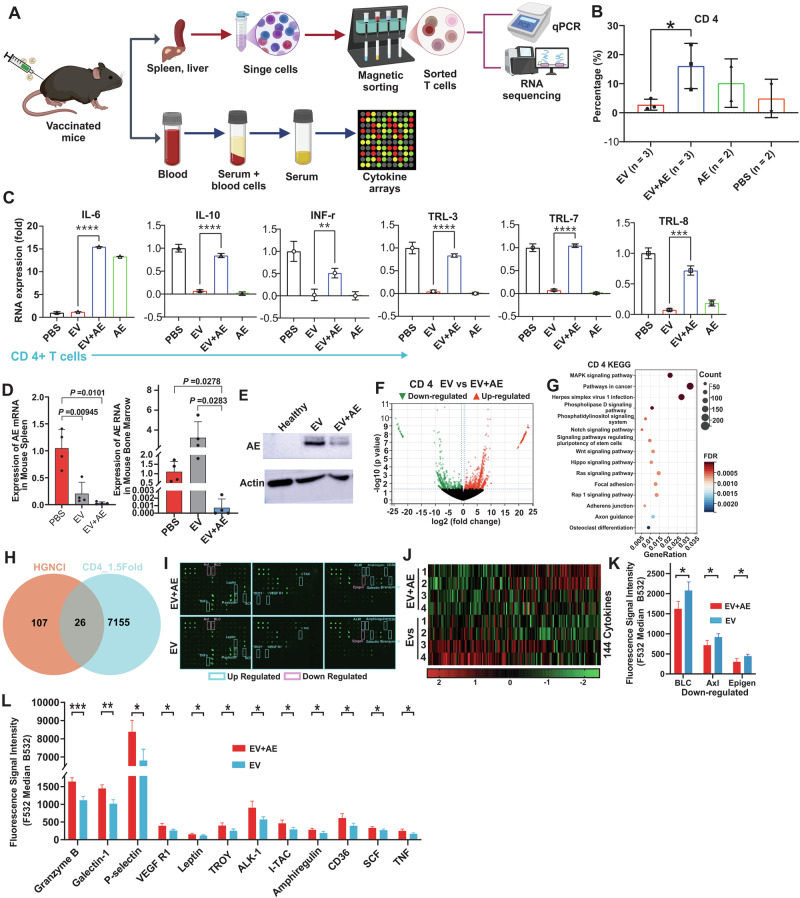
EV-encapsulated mRNA vaccination reduces leukemic burden via activating T cells with no obvious cytotoxicity. **A** Experimental design: Schematic illustration of the molecular characterization of organs from vaccinated and vehicle-treated mice. **B** The bar graphs show the percentage of CD4+ cells across different conditions. Splenic T cells were enriched from frozen single mouse splenic cells using a column-free magnetic separation method. Cells were then stained with antibody against the surface marker CD4. **C** qPCR measuring cytokine productions in CD4+ T-cell subsets. Splenic CD4+ T cells were enriched by magnet sorting. RNA levels of specific cytokines were quantified by qPCR. Data are presented as fold changes in expression, calculated using 2^−ΔΔCt^ method and normalized to β-actin. **D** Nest-qPCR to measure AE expression in splenic, and BM cells (*n* = 3 mice/group). **E** Representative Western blot showing AE protein expression in splenic cells. Sample sizes for Western blot analysis were *n* = 3 mice per group. **F**–**H** Leukemic C57BL/6 J mice were treated with EV-AE, EV or PBS over a total of five doses for 3 weeks. Two weeks after the final dose, spleens were collected, and single splenic cells were made. T cells with the immunophenotype CD4+ were isolated by magnet sorting for RNA sequencing (*n* = 3 mice/group). **F** Volcano plots show differentially expressed genes (log2 (fold change); ≥1.5 fold) comparing EV-AE-treated cells to EV-treated or PBS-treated cells. Red and blue dots indicate up- and down-regulated genes, respectively. **G** Bubble plots illustrate the top 15 enriched KEGG pathways for differentially expressed genes (log2 (fold change); ≥1.5 fold). The color of each bubble represents the adjusted FDR value, the size corresponds to the number of genes in the pathway, and the RichFactor indicates the proportion of genes in the key modules that belong to this pathway. **H** Venn diagram analysis illustrating overlap and differences of cytokine expression between our differentially expressed gene signature (log2 (fold change); ≥1.5 fold) and the published cytokine profile. **I** Scanned images from cytokine arrays. Each spot represents a pooled serum sample from four mice, with each group measured in quadruplicate. Red and green rectangles indicate significantly down- and up-regulated cytokines, respectively. **J** Heatmap showing the relative changes in 144 cytokines (*n* = 4 mice per lane). **K,** **L** Bars show the mean ± SD, and indicate the significantly up- or down-regulated cytokines (*P* < 0.05). The serum from 4 mice in each group was pooled and measured in quadruplicate. EV exosomes, AE AE mRNA in PBS, EV + AE AE mRNA delivered by EV, PBS leukemic mice injected with PBS as control, KEGG Kyoto Encyclopedia of Genes and Genomes, EV exosomes, EV-AE EV-delivered AE mRNA, SD standard deviation; ****P < 0.0001, ***P < 0.001, **P < 0.01, *P < 0.05; ns not statistically significant.

To evaluate the in vivo safety of AE-EVs, healthy C57BL/6 mice received five intramuscular injections of PBS, EVs, or EV-AE mRNA every four days (Fig. 1I). Histopathological examinations revealed no detectable pathological effects or damage in the heart, liver, spleen, lungs, kidneys, and skeletal muscle tissues of EV-AE vaccinated mice (Fig. 1J). No obvious changes were observed in body weight, mobility and capability of getting food and water. These findings indicate the absence of notable in vivo toxicity under these treatment conditions. To evaluate the in vivo therapeutic potential, C57BL/6 mice were injected with AE9a+ spleen cells, and treated intramuscularly with EVs only, AE only, LPN-AE, or EV-AE every four days for five doses (Fig. 1I). EV-AE treatment significantly reduced white blood cell (WBC) counts and improved survival in leukemic mice compared to controls (LPN-AE, EVs-only, AE-only, and PBS), which showed limited therapeutic benefit (Fig. 1K, L). H&E staining confirmed that EV-AE reduced leukemic infiltration and tissue damage in the spleen, lungs, and liver (Fig. 1M). No significant differences were observed in body/organ weights, or in kidney and muscle tissues (Fig. S2A, B). Furthermore, we observed that EV-AE treated mice exhibited significantly higher bone marrow (BM) dendritic cell (DC) counts than all other groups, including healthy and LPN-AE controls (*P* < 0.01; Fig. S3A, B). We compared treatments (EVs, AE mRNA, LPN-AE mRNA) to PBS control in leukemic mice. All treatments significantly enhanced immune functions, increasing splenic counts of CD4+ , CD8+ , CD11c+ , CD80+ , and MHCII+ cells (Figs. 2A, B and S3C, D). Specifically, EV-AE treatment strongly activated CD4 + T cells, showing a significant increase over the EV-only control (Fig. 2B). However, it did not significantly increase CD8+, MHCII+, or CD80+ counts relative to that control group (Fig. S3C). These findings suggest that while EV-AE promotes immunity via CD4+ T cell activation, its mechanism may not significantly involve boosting CD8+ T cells in the same manner as other treatments, potentially using a different pathway within the tumor microenvironment.

To assess the immune response to AE-EVs therapeutics, leukemic C57B/L6 mice were treated with PBS, EVs, EV-AE and AE mRNA. CD11c+ and CD8+ cells were magnet-sorted from spleens and analyzed via qPCR. As shown in Fig. S4A, B, in CD11c+ and CD8+ cells, the expression of IL-6, IFN-γ, IL-10, TLR-3 and TLR7, but not TLR-8, was significantly higher in all treated mice vs healthy and sick mice. AE-EVs treatment, compared to EVs alone, significantly upregulated IFN-γ, TLR-3 and TLR7 while downregulating IL-6, with no change in IL-10 or TLR-8, supporting AE-EVs’ specific effects. Further analysis of MHC-II+ cells showed marked increases in TLR-3, TLR-7, and TLR-8, and a significant decrease in IL-6 in AE-EVs-treated mice vs EVs alone or sick controls. No differences were observed in IL-10 and IFN-γ expression. Notably, TLR3 and TLR7 were significantly decreased in all treatment groups vs PBS. However, within the EV-AE group, TLR-7 was lower while TLR-3 remained unchanged compared to the EV-only group. TLR10 and IL-6 were undetectable across all groups.

To predict tumor regression accurately, we analyzed T-cell functionality focusing on T cell activation indicators (IFN-γ, IL-6, IL-10, TLR-3, TLR-7, and TLR-8). Compared to PBS and EVs controls, AE-mRNA immunization upregulated therapeutic indicators (IFN-γ, IL-10, and TLR-3/7/8) 2- to 3-fold, while downregulating the immunosuppressive cytokine IL-6 5- to 6-fold (Figs. 2C and S4C, D). The AE-mRNA vaccine recruits and activates DCs, CD4+ or CD8+ T cells in the blood, spleen, liver and BM, to initiate tumor clearance. Consequently, EV-AE-treated mice showed reduced AE+ blasts; qPCR confirmed decreased AE expression in the BM, and spleen (Fig. 2D), and Western blotting verified lower splenic AE protein levels (Fig. 2E). These results demonstrate that the AE mRNA vaccine induces targeted killing of AE+ AML cells and reduces organ infiltration.

To investigate T-cell activation by AE mRNA vaccination, we performed RNA sequencing (*n* = 3; Tables S1–4) on CD4+ and CD8+ T cells isolated from the spleens of leukemic mice treated with PBS, EVs, or EV-AE. Comparing EV-AE to PBS revealed over 18,242 transcripts in CD4+ and over 16,777 in CD8+ T cells. Contrastingly, EV-AE vs EVs showed over 17,336 transcripts in CD4+ and over 15,353 transcripts in CD8+ T cells. These differences were illustrated in volcano plot (Figs. 2F and S5A, B). Of the changes, 7658 transcripts were downregulated and 7140 upregulated compared EV-AE to PBS; 7183 were downregulated and 6525 upregulated compared EV-AE to EVs. Fold changes ranged from 1.5 to 25-fold or −22.5 to −1.5-fold. The full deregulated gene list is in Tables S5, 6. Heatmaps displayed the top 1,000 down- and upregulated genes (Fig. S5C, D).

GO and KEGG enrichment analyses of differentially expressed genes (DEGs) revealed that AE mRNA vaccination activates pathways essential for T-cell expansion and function. In CD4+ and CD8+ T cells, EV-AE enriched pathways related to RNA polymerase II regulation, immune signaling, and protein phosphorylation (Fig. S5E, F). KEGG analysis specifically highlighted MAPK, cytokine-cytokine receptor interaction, and cancer-related pathways (Figs. 2G and S5G, H), with MAPK and cancer pathways involving ~320 DEGs (Tables 5–S8). By comparing EV-only and EV-AE conditions, we focused on critical cytokine signaling. Overlapping RNA-seq data with a mouse cytokine profile identified 26 DEGs in CD4+ and 21 in CD8+ T cells (Figs. 2H and S5I; Table S9), most of which were shared. GO and KEGG analyses of these shared cytokines confirmed the engagement of immune responses, cell proliferation, and JAK-STAT, TNF, and PI3K-AKT signaling (Figs. S5J, K and S6).

While multiple pathways were identified, JAK-STAT and TNF signaling are particularly vital for antigen recognition and tumor clearance. JAK-STAT mediates immune regulation, while CD4+ -derived TNF promotes tumor-cell killing by recruiting CD8+ T cells and activating macrophages (Fig. S7; Table S10). Functional annotation via DAVID 6.8 identified IL-2, IL-3, IL-6, IL-10, and IL-15 as upstream activators of the JAK-STAT pathway (Fig. S8). Post-vaccination upregulation of STAT1, a Th1 differentiation regulator, confirmed activation across T-cell subsets. Furthermore, TNF binding to TNFR1 on AE+ AML cells was linked to altered IL-6 and IL-15 expression and RIP-mediated apoptosis (Figs. S9, S10). Collectively, these data suggest that TNF-overexpressing T cells target TNFR1-upregulated AML cells to induce TNFR1-dependent cell death.

In cancer vaccine therapy, serum cytokines are vital modulators of the immune response. We screened 144 serum analytes from EV- and EV-AE-treated leukemic mice using a cytokine antibody array (Fig. 2I; Table S11). The resulting heatmap revealed 80 upregulated and 64 downregulated factors (Fig. 2J). At a *P* < 0.05 threshold, 12 factors were significantly upregulated, including Granzyme B, Galectin-1, P-selectin, VEGF R1, Leptin, Troy, ALK-1, I-TAC, Amphiregulin, CD36, Kitl, and Tnf, while three (BLC, Axl, and Eplgen) were downregulated (Fig. 2K, L). GO and KEGG analyses of these 15 significant cytokines identified 96 affected functions, highlighting TNF signaling as a primary direct link, with MAPK and PI3K-AKT as indirect links (Fig. S11A, B). These results suggest that AE mRNA vaccination modulates systemic cytokine profiles and immune infiltration to coordinate tumor-immune communication via the MAPK, PI3K, and TNF pathways.

This study had limitations, including a small sample size, single-point immune measurements, and limited safety data. The vaccine showed potential against established leukemia, but preventive effects are unknown, and durability of the AE-specific immunity is unclear. The findings may extend to other fusion-driven cancers. Modest efficacy suggests combination therapy with inhibitors of AE partner proteins (e.g., HDACs) could be synergistic. Clinical relevance requires validation in humanized and AE9a knock-in models [15]. Future work must optimize mRNA structure (pseudouridine vs. unmodified) and conduct longitudinal studies (e.g., ELISpot) to capture dynamic immune activation and immune cell coordination. Despite these limitations, our findings demonstrate that targeting AE chimeric genes yields potent anti-tumor effects. This strategy enhances immunogenicity and offers a promising path for non-personalized mRNA vaccines against fusion-driven malignancies.

## Supplementary information

Supplementary Materials

Dataset 1

Dataset 2

Dataset 3

Dataset 4

Dataset 5

Dataset 6

Dataset 7

Dataset 8

Dataset 9

Dataset 10

Dataset 11

## Data Availability

All sequencing data generated in this paper can be accessed from ERP185178 (European Nucleotide Archive, ENA) and are publicly available as of the date of publication. Accession numbers are listed on the key resources table. All other data are available upon request from the lead contact/corresponding author.
