# Supplementary material for: mRNA vaccination targeting AML1::ETO fusion gene eliminates leukemia cells via activating T cells
Source: Leukemia. 2026 Apr 10;40(6):1335–9. doi: 10.1038/s41375-026-02940-3 (PMC13233333; doi:10.1038/s41375-026-02940-3)
Supplement: Supplementary file 1 — Supplementary Materials [file 41375_2026_2940_MOESM1_ESM.docx]

**Supplementary Information**

**mRNA Vaccination Targeting AML1/ETO Fusion Gene Eliminates Leukemia Cells via Activating T Cells**

Changli Zhou, Sicheng Bian, Jiuxia Pang, Huiqin Bian, Tao Cheng, Hiroaki Koyama, Bin Liu, Bing Li, William Tse, Shujun Liu

**Inventory of Supplemental Information**

**Supplemental Methods**

**Supplemental Figures and Legends**

**Supplemental Tables**

**Methods**

**Mice**

C57BL/6 mice (4–6 weeks old, male and female) were purchased from the Jackson Laboratory. All animal procedures were performed according to NIH guidelines and approved by the Institutional Animal Care and Use Committee at the University of Minnesota. Mice were monitored daily for signs of deteriorating health, as indicated by weight loss, slow movement, or hunched posture. All mice had free access to food and water throughout the study.

**Cell lines and cell culture**

The HEK293 cell line, newly purchased from American Type Culture Collection, was cultured in DMEM (GE Healthcare, #SH30027.01) supplemented with 10% fetal bovine serum (FBS, Gibco by Life Technologies^TM^, #16140-071) and Antibiotic-Antimycotic (Gibco by Life Technologies^TM^, #15240062) at 37 °C under 5% CO_2_. The cell line was not independently authenticated or tested for mycoplasma, but it is not listed in the International Cell Line Authentication Committee (ICLAC) database of commonly misidentified cell lines.

**Retrovirus vector, virus production, virus infection and GFP sorting**

For virus production, HEK-293 (3.8 × 10^6^) cells were planted in a 10 cm cell culture dish for 24 hours, and transfected with 6 µg of targeted or scrambled plasmids using calcium phosphate transfection reagent (CalPhos™ Mammalian Transfection Kit, TakaraBio, # 631312), following the manufacture’s instruction. The retroviruses were harvested at 48 and 72 hours after transfection and concentrated using the protocol of the Lenti-X™ Concentrator (Clotech, #631232). For virus infection, HEK-293 cells (1 × 10^6^) were infected by the retroviruses using Polybrene (Sigma-Aldrich, # TR-1003) (final concentration 4 µg/ml) in 1 ml medium. The GFP positive cells were sorted at 72 hours post-infection, expanded and sorted again for further investigations.

**EV purification from** **red blood cells (RBCs)**

Exosomes (EVs) were purified from RBCs as previously reported.(*80*) Briefly, RBCs were treated with 10 µM calcium ionophore (Sigma Aldrich, #A23187) overnight. To purify EVs, RBCs and cell debris were removed by centrifugation at 600 × g for 20 min, 1600 × g for 15 min, 3260 × g for 15 min, and 10,000 × g for 30 min at 4 °C. The supernatants were passed through 0.45 μm-syringe filters. EVs were concentrated by ultracentrifugation in a Beckman XE-90 ultracentrifuge with a TY50.2Ti rotor (Beckman Coulter) at 56,000 × g for 24 hours at 4 °C, and resuspended in cold PBS. Purified EVs were stored at −80 °C.

**Staining and visualization of exosomes by Nanoimager**

The exosomes (EVs) were visualized using the super-resolution microscope Nanoimager (ONI) with the NimOS software following immunofluorescent labeling of exosomal surface markers. Briefly, the isolated EVs were incubated with fluorescently labeled primary antibodies targeting specific tetraspanin proteins (CD63, CD81, or CD9) following the manufacturers’ instructions. The specific fluorophore conjugated to each antibody (e.g., Alexa Fluor 647, Cy3) was selected to be compatible with the Nanoimager's laser lines. Unbound antibodies were removed through multiple washing steps to reduce background fluorescence and ensure a high signal-to-noise ratio. The NimOS software was used for image acquisition. All comparable images were acquired using identical settings, including laser power, exposure time, and gain, to ensure data consistency and reproducibility.

**Negative staining procedure for exosome visualization**

The morphology and size distribution of exosomes were analyzed using negative staining transmission electron microscopy (TEM). Four microliters of the sample solution (~0.3 μM) were applied to freshly glow-discharged, 200-mesh carbon-coated copper grids (EM Sciences) and incubated for 1 minute. After the 60-second incubation, excess liquid was removed by blotting the grid's edge with filter paper. This was followed by three successive washes using droplets of deionized water to remove residual buffer salts, which can interfere with staining. The washed grid was then incubated for 30 seconds in a 0.75% uranyl formate solution. Excess stain was removed by blotting the grid's edge. The grid was then allowed to air-dry completely at room temperature before being loaded into the microscope. Imaging was performed on a Biotwin Tecnai Spirit 120 kV electron microscope (ThermoFisher Scientific) equipped with a Gatan 4K × 4K CCD camera, at a defocus of -2 μm and nominal magnifications ranging from 18,500 × to 98,000 × to ensure optimal visualization across different scales.

**RNA constructs, *in vitro* transcription, and purifications**

Full length AE9a gene was amplified from MigR1-AE9a by PCR using High-Fidelity 2× Master Mix (NEB, #M0492). The primers for PCR include:

pGEM4z-Frag01_fwd aggatgaAGCGGCCGCGGATCCCCG

pGEM4z-Frag01_rev tacgggaCACCATGGTGGCGACCGGT

AE9a-Frag02_fwd ccatggtgTCCCGTATCCCCGTAGA

AE9a-Frag02_rev cggccgctTCATCCTAGTGCAACTG

The fragments were cloned into the multiple-cloning site (MCS) of T7 promoter plasmids pGEM4z-GFP-64A using Gibson Assembly Cloning Kit (New England Biolabs, #E5510S). The generated pGEM4z-AE9a-664A vector was confirmed by sequencing. The 3′ end poly(A) tail and 5′ end was modified. The modifications of 3′ end include a 63-bp poly(A) tail and two serial fragments (UTR) in front of the poly(A) tail.

For *in vitro* mRNA transcription from the DNA template, the plasmid pGEM4z-AE9a-64A was linearized by SpeI-HF (New England Biolabs, #R3133S), the linearized pGEM4z-AE9a-64A plasmid was used as a template. The *in vitro* mRNA transcription was performed using HiScribe™ T7 High Yield RNA Synthesis Kit (New England Biolabs, # E2040S). The 5’CAP is S1411L 3´-O-Me-m7G(5')ppp(5')G RNA Cap Structure Analog. The uridine-5’-triphosphate (UTP; TriLink, #N1504) was 100% replaced by 1-methylpseudouridine-5’-triphosphate (m1ѰTP) (TriLink, #N-1081). The reactions were incubated at 37 °C for 2 hours. Then the DNase I (RNase-free; New England Biolabs, #M0303L) was added and incubated at 37 °C for 15 min to remove template DNA. mRNA quality control was performed by spectrophotometry on a 2100 Bioanalyzer (Agilent technologies).

The dsRNA in 100 to 500 mg IVT mRNA was removed as previously described by using microcentrifuge spin columns (NucleoSpin Filters, Macherey-Nagel, Düren, Germany), cellulose fibers (SigmaAldrich, #C6288), and a chromatography buffer containing 10 mM HEPES (pH 7.2), 0.1 mM EDTA, 125 mM NaCl, and 16% (v/v) ethanol as previously described. Finally, we used Monarch® RNA Cleanup Kit (500 μg; New England Biolabs, #T2050) to clean the mRNA, electrophoresis to determine RNA quality and stored mRNA in pure water at -80^o^C.

**Preparation and characterization of mRNA-encapsulated EVs**

The AE-EVs were prepared using Exo-Fect™ Exosome Transfection Reagent (System Biosciences, #EXFT20A-1) with the manufacturer’s protocol. In brief, the transfection reaction was performed by combining 10 µl Exo-Fect solution, 5 µl mRNA (10 µg), 133 µl sterile 1 × PBS with 2 µl purified exosomes (2 × 10^8^) in a 1.5 ml tube achieve a total reaction volume of 150 µl. The components were mixed well by flicking/ inversion three times. Vortexing was avoided to prevent damage to the exosomes. The mixture was incubated at 37°C in a shaker for 10 min before being immediately transferred to ice. To terminate the reaction, 30 µl of the ExoQuick-TC reagent was added to the samples and mixed by inverting 6 times. The transfected exosomes were placed on ice (or at 4°C) for 30 minutes, and then centrifuged for 5 minutes at 13,000-14,000 rpm in a microcentrifuge (top speed). The supernatant was removed, and the transfected exosome pellets were resuspended in 150 µl 1 × PBS.

**RNA loading efficiency and stability in EVs**

To quantify the amount of unbound AE9a mRNA, 2 µg of mRNA were uploaded into EVs (1 × 10^7^) using Exo-Fect™ Exosome Transfection Reagent (System Biosciences, #EXFT20A-1) with the manufacturer’s protocol. Following centrifugation, the mRNA was precipitated from the supernatant, and its concentration was measured with a NanoDrop spectrophotometer. To visualize the mRNA, an equivalent amount was separated by electrophoresis on a 10% Tris-acetate-EDTA (TBE) native polyacrylamide gel at 150 V for 30 minutes and subsequently stained with SYBR-Gold (ThermoFisher Scientific, #S11494).

***In vitro* transfection of HEK293 cells by** **AE-mRNA**

HEK293 cells were cultured at a density of 5 × 10^4^ cells/well in DMEM supplemented with 10% FBS overnight in 35-mm dishes. Cells in the logarithmic growth phase (a confluency of approximately 70–80%) were transfected with AE-mRNA delivered by different vehicles. In EV-AE group: approximately 150 µl of transfected exosomes was added to 10^5^ cells per well in a 6-well culture plate containing in opti-MEM media. The exact volume and concentration were scaled as required by specific experimental parameters, based on an optimized exosome-to-cell ratio. The experiment groups included EVs only, AE-mRNA only, Lipofectamine 2000-AE-mRNA, and the EV-AE-mRNA group (EV-AE). In Lipofectamine 2000-AE-mRNA group: as a positive control for conventional lipid-mediated transfection, AE-mRNA was complexed with Lipofectamine 2000 following the manufacturer's protocol. The transfection complexes were prepared in Opti-MEM and then added to the cells. In AE-mRNA only group: naked AE-mRNA was added directly to the cell culture as a negative control to assess the effect of free mRNA delivery. In EVs only group: Isolated, untransfected exosomes were added to cells to serve as a negative control for any effects mediated by the EVs themselves. After transfection, the cells were incubated at 37°C for a predetermined period ranging from 2 to 24 hours. The incubation time was optimized to capture peak expression of the reporter protein while minimizing cytotoxicity. Following incubation, the cells were subjected to analysis using fluorescent microscopy for GFP expression or harvested for Western blot to validate target protein expression.

**RNA isolation and quantitative PCR (qPCR)**

Total RNA was isolated from various types of cells using the RNeasy Mini Kit (Qiagen, #74104), following the manufacturer's recommended protocol. First-strand cDNA was synthesized from the isolated total RNA using the SuperScript® III First-Strand Synthesis System (Invitrogen, #18080051). Expression of AE was detected using the TaqMan® Gene Expression Assay (Applied Biosystems, #4331182). The expressions of INF-γ, IL-6, IL-4, IL-10, TLR3, and other genes were measured using the Power SYBR® Green PCR Master Mix (Applied Biosystems, #4367659). Relative gene expression levels for all target genes were determined using the comparative cycle threshold (Ct) method. All data were normalized against the expression levels of the housekeeping gene GAPDH. The primers are listed in Supplementary Table 12.

**Western blotting**

The whole cellular lysates were prepared by harvesting the cells in 1 × cell lysis buffer (20 mM HEPES (pH 7.0), 150 mM NaCl and 0.1% NP40) supplemented with 1 mM phenylmethane sulfonyl fluoride (PMSF; Sigma, #10837091001), 1 × Phosphatase Inhibitor Cocktail 2 and 3 (Sigma, #P5726, #P0044), and 1 × protease inhibitors (protease inhibitor cocktail set III; Calbiochem-Novabiochem, #539134). Proteins were resolved by sodium dodecyl sulfate (SDS)–polyacrylamide gel electrophoresis, transferred onto PVDF membranes (GE Healthcare, #10600023). The membranes were blocked for 1 hour at room temperature in Tris-buffered saline with 0.1% Tween 20 (TBST) buffer containing 5% non-fat milk. After blocking, the membranes were incubated with primary antibodies (anti-GFP, anti-β-Actin, anti-AML1/ETO) diluted in the blocking buffer for overnight at 4°C. The membranes were then washed three times for 10 minutes each with TBST, and incubated for 1 hour at room temperature with the secondary antibodies (horse anti-mouse IgG, goat anti-rabbit IgG, or rabbit anti-goat IgG). Finally, the membranes were developed using an enhanced chemiluminescence substrate following manufacturer's instructions and the resulting signal was captured with an imaging system.

**Local administration of mRNA-exosome/lipofectamine complex**

C57BL/6 mice (4-6 weeks; male, female) were purchased from Jackson Laboratory. All mice were maintained under specific pathogen-free conditions. All animal experiments were performed in strict accordance with the recommendations in the Guide for the Care and Use of Laboratory Animals of the US National Institutes of Health. The protocols were reviewed and approved by the Institutional Animal Care and Use Committee (IACUC) of the University of Minnesota. Mice were euthanized and sacrificed using CO_2_ as per the institutional guidelines in all experiments, when they showed any signs of distress (e.g., breathing disorders, weight loss, immobility or oversized tumors), for tissue collections and survival duration.

About 5 × 10^4^ AE9a liver cells were injected into C57BL/6 mouse via the tail veil. Five days post-tail injection of leukemia cells, intramuscular (IM) injections of mRNA delivered by various vehicles into the musculus gastrocnemius were performed and repeated every 4 days, for a total of five injections. Before the injection, the injection site on the hind leg was shaved to improve visibility of the target muscle and to reduce the risk of introducing contaminants. The injection site was then disinfected with an alcohol swab. For each injection, 75 µl of either exosome (EV), AE mRNA (AE in PBS), AE mRNA-exosome (EV-AE), AE mRNA-Lipofectamine (LPN-AE) -solution or PBS control was administrated. Mice were humanely sacrificed 3 weeks after the final intramuscular injection. Blood, bone marrow (BM), liver, and spleen were collected from the sacrificed mice. Plasma was isolated from the blood, and both the plasma and bone marrow were stored at −80°C for future analysis. Cytospin preparations of BM cells were processed for Giemsa staining to analyze cell morphology, differential cell counts, and the overall state of the hematopoietic lineage. Parts of livers and spleens were used to generate single cell suspensions.

**Hematoxylin and eosin (H&E) and Immunohistochemistry (IHC) staining**

Tissue specimens (lungs, spleen, kidneys, heart, muscle, and liver) were harvested from animals and immediately stabilized by immersion in a 10% neutral buffered formalin solution. The paraffin-embedded samples were then processed by the Department of Pathology at the MetroHealth Medical Center for standard H&E and IHC staining.

**Cytospin/Wright-Giemsa staining**

About 0.1 × 10^6^ BM cells were harvested from vaccinated mice and placed in Shandon EZ Single Cytofunnel (Thermo Electron Corporation). Samples were centrifuged at 800 rpm for 3 min. The slides were air-dried and stained using Hema-3 Kit (Fisher Scientific, #23-123869). Stained slides were viewed and photographed using a Leica microscope mounted with a high-resolution spot camera with Image-Pro Plus software. Morphologic differentiation was determined by calculating the percentage of post-mitotic cells containing metamyelocytes, bands and segmented neutrophils within six visual fields per slide.

**Characterization of immune cells by magnetic or flow cell sorting**

After vaccination, spleens or livers were isolated and prepared into single cell suspension. Spleens were mashed through a 70-µm cell strainer (BD-Falcon) using the plunger of a 5-ml syringe (BD Biosciences) while rinsing with PBS. Erythrocytes were removed by hypotonic lysis. Fluorescence-activated cell sorting (FACS) surface and intracellular antibodies were purchased from eBioscience, Bioledgend or BD Pharmingen and used in accordance with the manufacturer’s protocol. Single cell suspensions were stained for 30 min at 4 °C for extracellular markers. All cell-based analyses were performed on single cell suspensions. Immune cells from spleens or liver were subjected to surface and intracellular staining. Flow cytometric data were collected with a BD FACS Calibur™. Single immune cell population from spleens or liver was separated with a BD FACSAria II Cell Sorter. Flow cytometric data were analyzed with FlowJo software.

Regarding the magnet sorting, the single cells were incubated with microbeads coated by antibodies against CD11c, CD4, CD8, CD80 or MHCII (CD74). The respective positive cells were isolated using L3T4 microbeads and MACS LS columns (Miltenyi Biotec) as manufacturer’s recommendations. The reagent kits are: EasySep Release Mo APC Pos Slctn Kit (STEMCELL Technologies, #100-0033), EasySep Mouse CD4+ T Cell Isolation Kit (STEMCELL Technologies, #19852), EasySep Mouse CD8+ T Cell Isolation Kit (STEMCELL Technologies, #19853) and EasySep™ Mouse CD11c Positive Selection Kit II (STEMCELL Technologies, #18781RF).

**Measurement of serum cytokine concentrations by cytokine arrays**

Blood samples were collected from leukemic mice that were treated with either Exo or Exo+AE mRNA. The samples were drawn into serum collection tubes and allowed to clot at room temperature for approximately 30 minutes. Following this, the tubes were centrifuged at 3,000g at 4 ^o^C for 5 minutes to separate the serum. The resulting supernatants were carefully transferred to individual polypropylene tubes and immediately stored at –80 °C for until further analysis.

For the simultaneous, multiplexed quantitation of cytokines and chemokines, four individual mouse serum samples were pooled to form a single representative sample. The pooled sample was then analyzed using the RayBio® Mouse Cytokine Antibody Array G-Series 2000 (RayBiotech Life; #AAM-CYT-G2000-4 containing 144 cytokines and chemokines) according to the manufacturer's instructions. The immunoassay was performed in quadruplicate to ensure high precision and reproducibility. The captured immunoassay signals were then read and quantified using the Array Scanning and Analysis Services offered by RayBiotech Life. The average of the quadruplicate readings was used to calculate the final concentration for each cytokine in the serum. Statistical differences in measured values were analyzed using unpaired t test with welch correction. *P*-values were calculated based on the four signal outputs from the quadruplicate assays. A p-value of less than 0.05 was defined as statistically significant, and no fold-change cut-off was applied. All data are presented and a complete list of the 144 cytokines and chemokines measured can be referenced in the corresponding reagent kit's manual.

**Immune monitoring with bulk RNA sequencing**

The effects of mRNA-Exo versus Ctrl on immune cells were investigated by RNA sequencing for the expression of immune-relevant factors (e.g., cytokines). The splenic cells were incubated with antibodies against CD4+ or CD8+, and sorted by magnet isolation. Total RNA was extracted from these CD4+ or CD8+ T cells using RNeasy Mini Kit (Qiagen #74104). The strand specific transcriptome library construction was completed by enriching mRNA from total RNA and sequenced by DNBSEQ high-throughput platform at BGI (https://www.bgi.com). Briefly, mRNA molecules were purified from total RNA using oligo(dT)-attached magnetic beads and fragmented into small pieces using fragmentation reagent after reaction a certain period in proper temperature. First strand cDNA synthesis was done by adding appropriate amounts of primers to the interrupted sample, mixing well, and reacting at a suitable temperature on a thermal cycler for a certain period of time to open the secondary structure and combine with the primers. Then the second strand cDNA synthesis was done by preparing a second-strand synthesis reaction system (using dUTP instead of dTTP). The second-strand cDNA was purified by magnetic beads. The end repair & add ‘A’ was done by repairing the sticky ends of the cDNA double-stranded by reverse transcription and add A base to the 3'end. Adaptor ligation was done by connecting the linker to the A base, and the reaction product was purified by magnetic beads. PCR amplification was done by digesting the U-labeled second-strand template with UDG enzyme. PCR products were purified with XP Beads, and dissolved in EB solution. The library was validated on the Agilent Technologies 2100 bioanalyzer. The double stranded PCR products were heat denatured and circularized by the splint oligo sequence. The single strand circle DNA (ssCir DNA) were formatted as the final library. The library was amplified with phi29 to make DNA nanoball (DNB) which had more than 300 copies of one molecular. The DNBs were loaded into the patterned nanoarray, and single end 50 (pair end 100/150) bases reads were generated in the way of combinatorial Probe-Anchor Synthesis (cPAS). Triplicate samples were sequenced.

Data were then processed, and basic quality metrics were checked using Illumina Genome Studio. For a gene to be considered significantly up- or down-regulated, the fold difference is ≥1.5 (log2 (fold change)). This “cutoff” was used for further analysis, including the signaling pathway by GO or DAVID bioinformatics resources (Version 6.7; https://david.ncifcrf.gov/tools.jsp). All images were made online using Sangerbox 3.0 (http://vip.sangerbox.com/home.html). The FDR or *P* values of ≤0.05 were used to select top signaling pathways and genes involved in for further verification.

**Statistical analysis**

Statistical analyses were performed using GraphPad Prism 5. All graphs were generated with the Student's t-test, and Kaplan-Meier survival curves were created using the log-rank test. determined based on literature documenting similar, well-characterized experiments, ensuring they were sufficient for statistical analysis of experimental versus control outcomes. *In vitro* experiments, such as qPCR, cell proliferation assays, and clonogenic assays, were routinely repeated three times unless otherwise noted in the figure legends or main text. All *P* values were two-tailed. No samples or animals were excluded from the analysis. All criteria were pre-established. No randomization was used in our studies. No blinding for all experiments. Variations were compatible between groups and the chosen statistical tests were justified as appropriate for each figure. Significance was determined at **P* <0.05, ***P* <0.01 or ****P* <0.001.


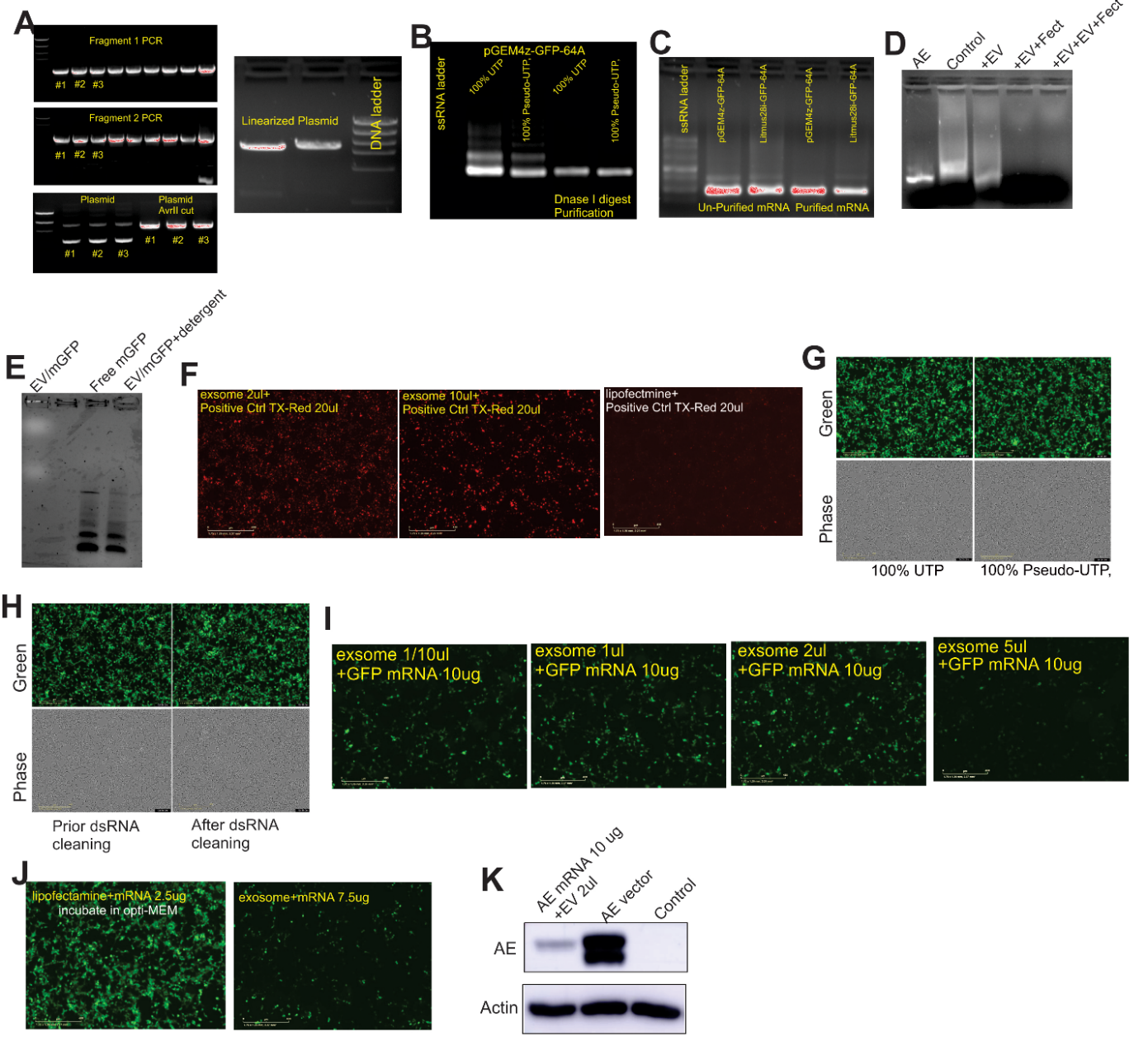


**Supplementary Figure 1.** **Characterization of mRNA encapsulated EV.** **A** Agarose gel analysis of AE9a mRNA expression vector driven by T7 promoter, indicating the success of AE9a cloning (left) and the *in vitro* synthesized GFP mRNA. The gel at lower panel confirmed successful cloning by restriction enzyme digestion. **B, C** Agarose gel analysis of the synthesized AE9a and GFP mRNA with or without contamination of dsRNA and plasmid template DNA. **D** Agarose gel analysis of AE9a mRNA within or being released from EV. **E** Agarose gel analysis of mRNA released from the broken EVs verifying the success of EV uploading. The data are representative of 3 independent experiments. **F**-**J** 293T cells were transfected with GFP mRNA (with or without Pseudo-UTP). The expression of GFP protein was captured by fluorescent imaging. The data are representative of three independent experiments. **K** Western blot analysis of AE protein expression in 293T cells. Cells were transfected with AE expression DNA plasmids via LPNs or with AE expression mRNA delivered by EVs. The data shown are representative of three independent experiments. **Abbreviations:** EV, exosomes; dsRNA, double strand RNA; AE, AE mRNA; mGFP, GFP mRNA; LPNs, liposome nanoparticles.

**Supplementary Figure 2.** **Histopathological examination of organs from healthy and treated mice.** **A** Bars are the comparison of body and organ weights in mRNA vaccinated mice vs healthy controls. **E** Representative images of the spleen, lung, liver, muscle, heart, and kidney from healthy and vaccinated leukemic mice (n = 3 mice/group). **Abbreviations:** ns, no statistically significant; EV, exosomes; LPN, lipofectamine nanoparticles; AE, AE mRNA in PBS; EV+AE, AE mRNA delivered by EV; LPN-AE, AE mRNA delivered by LPN.
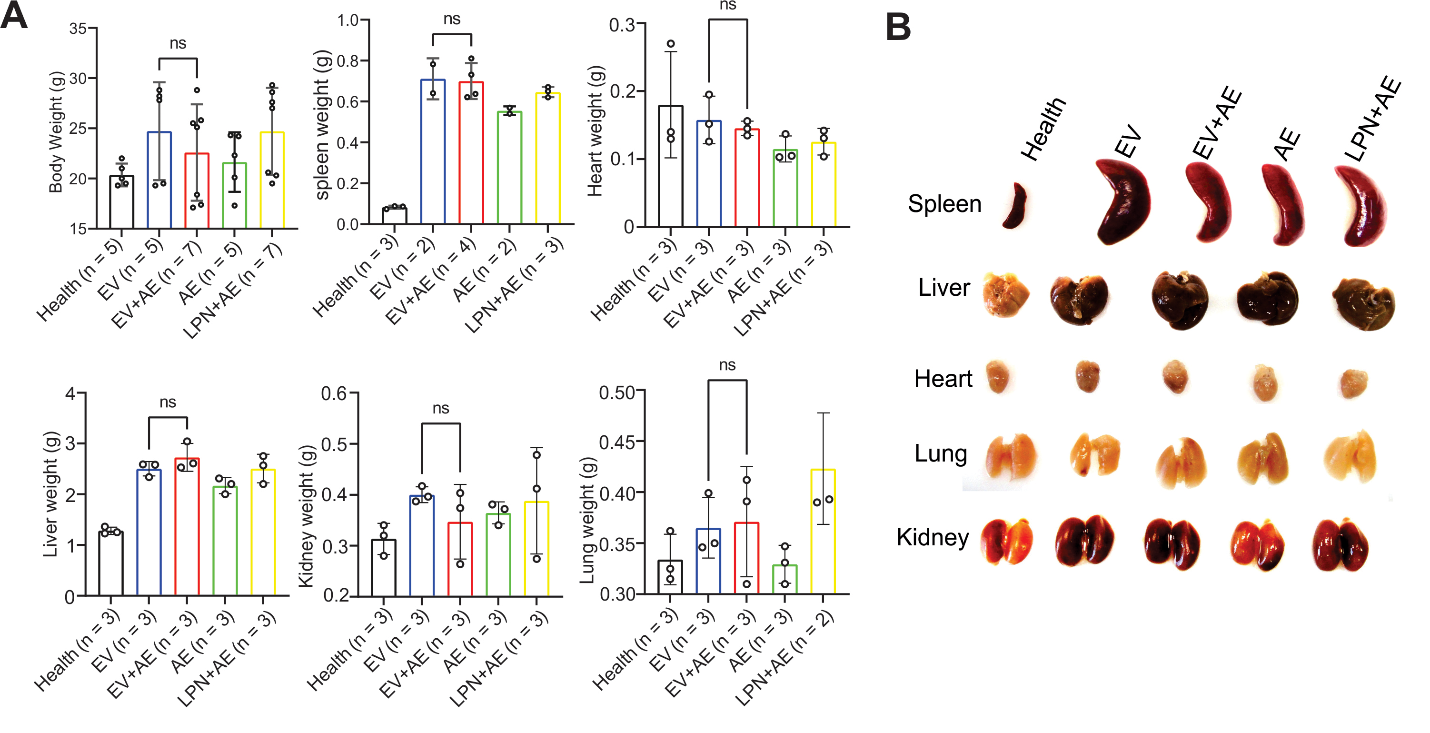


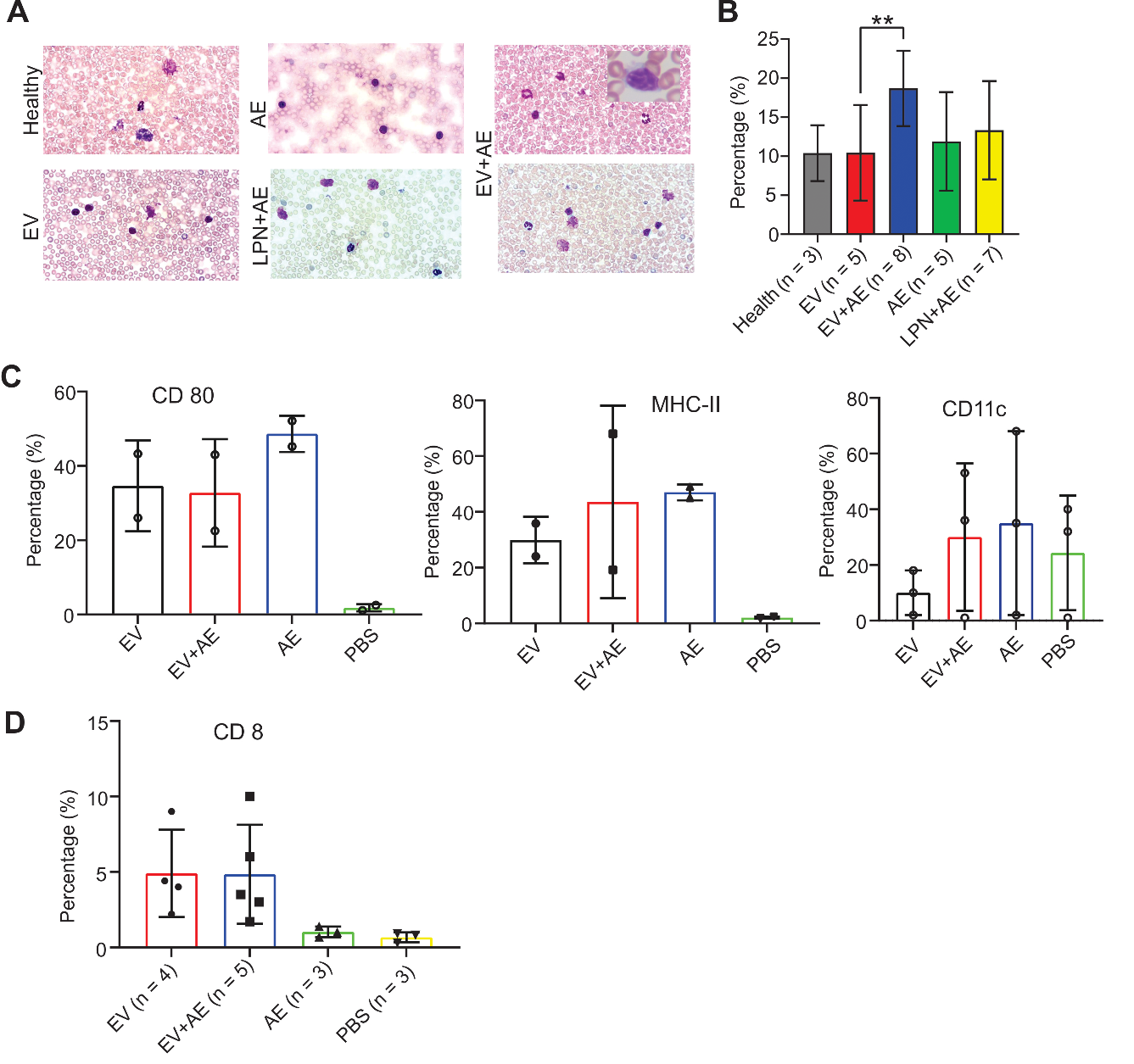
**Supplementary Figure 3.** **Treatment with EVs-delivered AE mRNA ameliorates immune cells in mice. A** Representative image of Wright-Giemsa-stained blood smears from leukemic mice treated with different mRNA vaccinations. The enlarged image at right upper corner highlights the dendritic cell-like cells, which appear more frequent in EV-AE than other groups. The images represent multiple mice in each group: healthy (n = 3), EV (n = 5), EV-AE (n = 8), AE mRNA only (n = 5), and LPN-AE (n = 7). **B** Quantification of dendritic cell-like cell frequency from blood smears across the different treatment groups. **C**, **D** The bar graphs show the percentage of CD8+, CD80+, CD11c+, MHC-II+ cells across different conditions. Splenic T cells were enriched from frozen mouse splenic cells using a column-free magnetic separation method from frozen cell stocks. Cells were then stained with antibodies against the surface markers CD4+, CD8+, CD80, CD11c, or MHC-II. **Abbreviations:** EVs, exosomes; LPN, lipofectamine nanoparticles; AE, AE mRNA only. EV, exosomes; AE, AE mRNA in PBS; EV+AE, AE mRNA delivered by EV; PBS, leukemic mice injected with PBS as control; **P* < 0.05; ***P* <0.01.


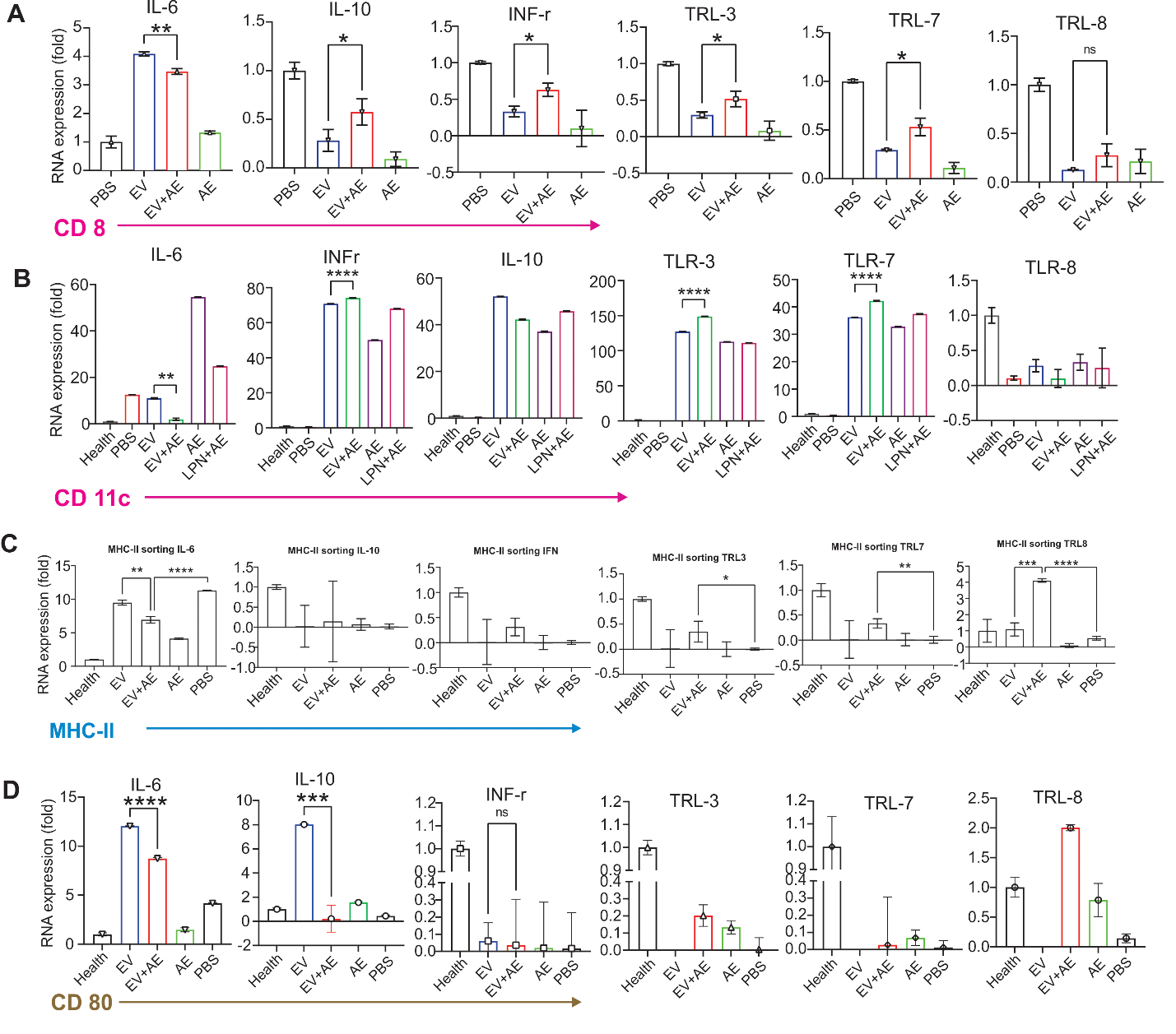
**Supplementary Figure 4.** **Cytokine expression in immune cells isolated from mRNA vaccine treated mice.** **A**-**D**. CD8+, CD11c+, MHC-II+ and CD8+ splenic cells were isolated by magnetic sorting. The extracted RNA from these immune cells was subjected to qPCR. **Abbreviations:** **P* <0.05, ***P* <0.01, ****P* <0.001, *****P* <0.0001. EV, exosomes; EV+AE, AE mRNA delivered by EV; LPN, lipofectamine nanoparticles; AE, AE mRNA only; LPN+AE, mRNA delivered by lipofectamine nanoparticles; PBS, leukemic mice injected by PBS.


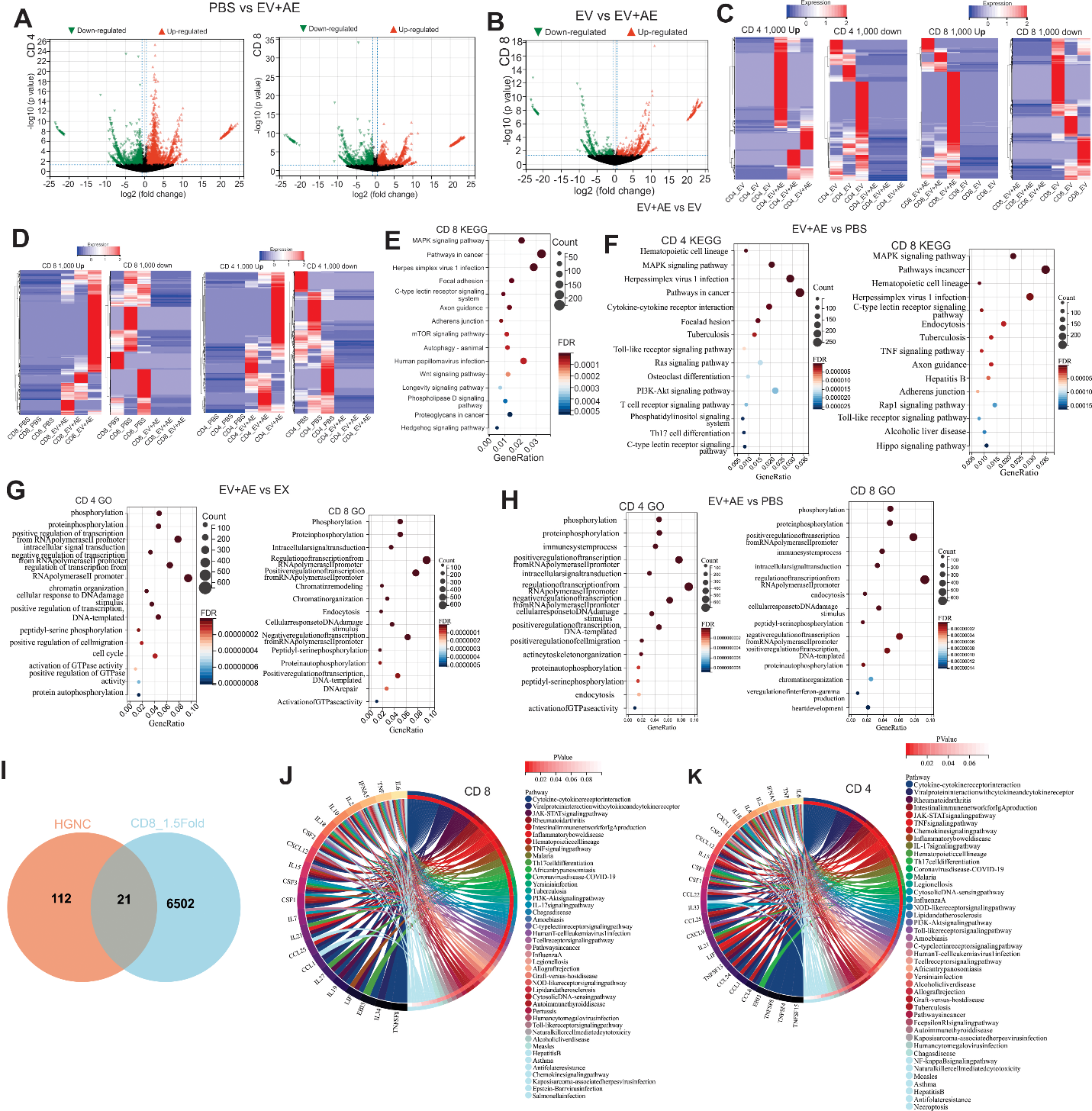
**Supplementary Figure 5.** **Bulk RNA-Seq of splenic CD8+ and CD4+ T cells from C57BL/6 mice treated with EV-AE, EV or PBS.** Leukemic C57BL/6 mice were treated with EV-AE, EV or PBS over a total of five doses for 3 weeks. Two weeks after the final dose, spleens were collected, and single splenic cells were made. T cells with the immunophenotype CD4+ or CD8+ were isolated by magnet sorting for RNA sequencing (n = 3 mice/group).

**A**, **B** Volcano plots show differentially expressed genes (log2 (fold change); ≥1.5 fold) comparing EV-AE-treated cells to EV-treated or PBS-treated cells. Red and blue dots indicate up- and down-regulated genes, respectively. **C**, **D** Heatmaps display the top 1,000 up- and down-regulated genes (log2 (fold change); >1.5 fold). **E**, **F** Bubble plots illustrate the top 15 enriched KEGG pathways for differentially expressed genes (log2 (fold change); ≥1.5 fold). The color of each bubble represents the adjusted FDR value, the size corresponds to the number of genes in the pathway, and the RichFactor indicates the proportion of genes in the key modules that belong to this pathway. **G** Venn diagram analysis illustrating overlap and differences of cytokine expression between our differentially expressed gene signature (log2 (fold change); ≥1.5 fold) and the published cytokine profile. **H**, **I** KEGG circle network plot (left) showing a chord dendrogram of the clustering of the expression spectrum of significantly changed cytokines derived from “**G**”. The names of the top KEGG signaling pathways are marked on the right of the chord diagram, the adjusted *P* values are marked with different colors. **Abbreviations:** KEGG, Kyoto Encyclopedia of Genes and Genomes; EV, exosomes; EV-AE, EV-delivered AE mRNA.


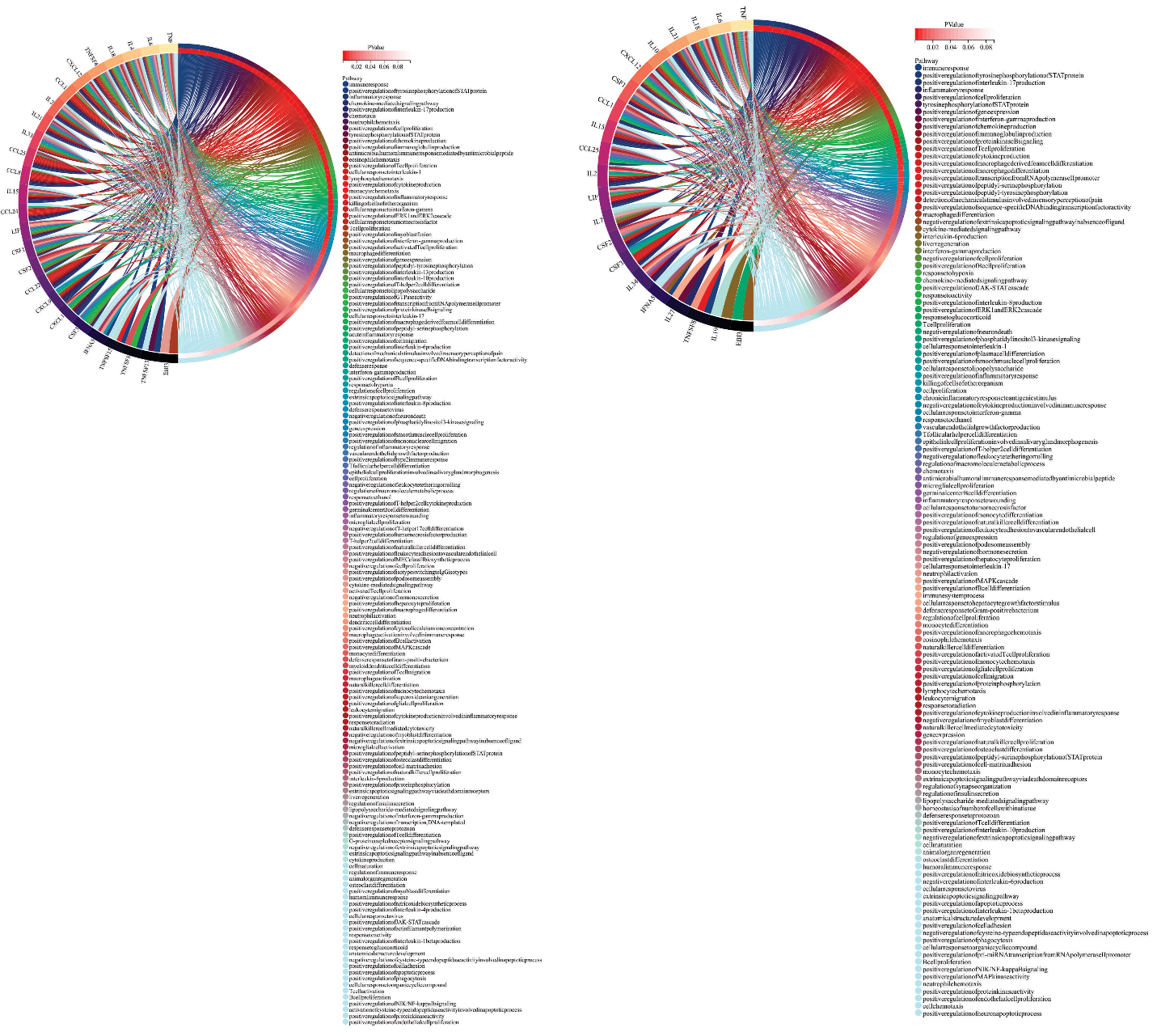
**Supplementary Figure 6.** **GO enrichment analysis for differentially expressed cytokines.** The circle network plot (left) shows a chord dendrogram of the clustering of the expression spectrum of significantly changed cytokines (log2 (fold changes); ≥1.5 or ≤1.5). The names of the top GO signaling pathways are marked on the right of the chord diagram, the adjusted *P* values are marked with different colors.


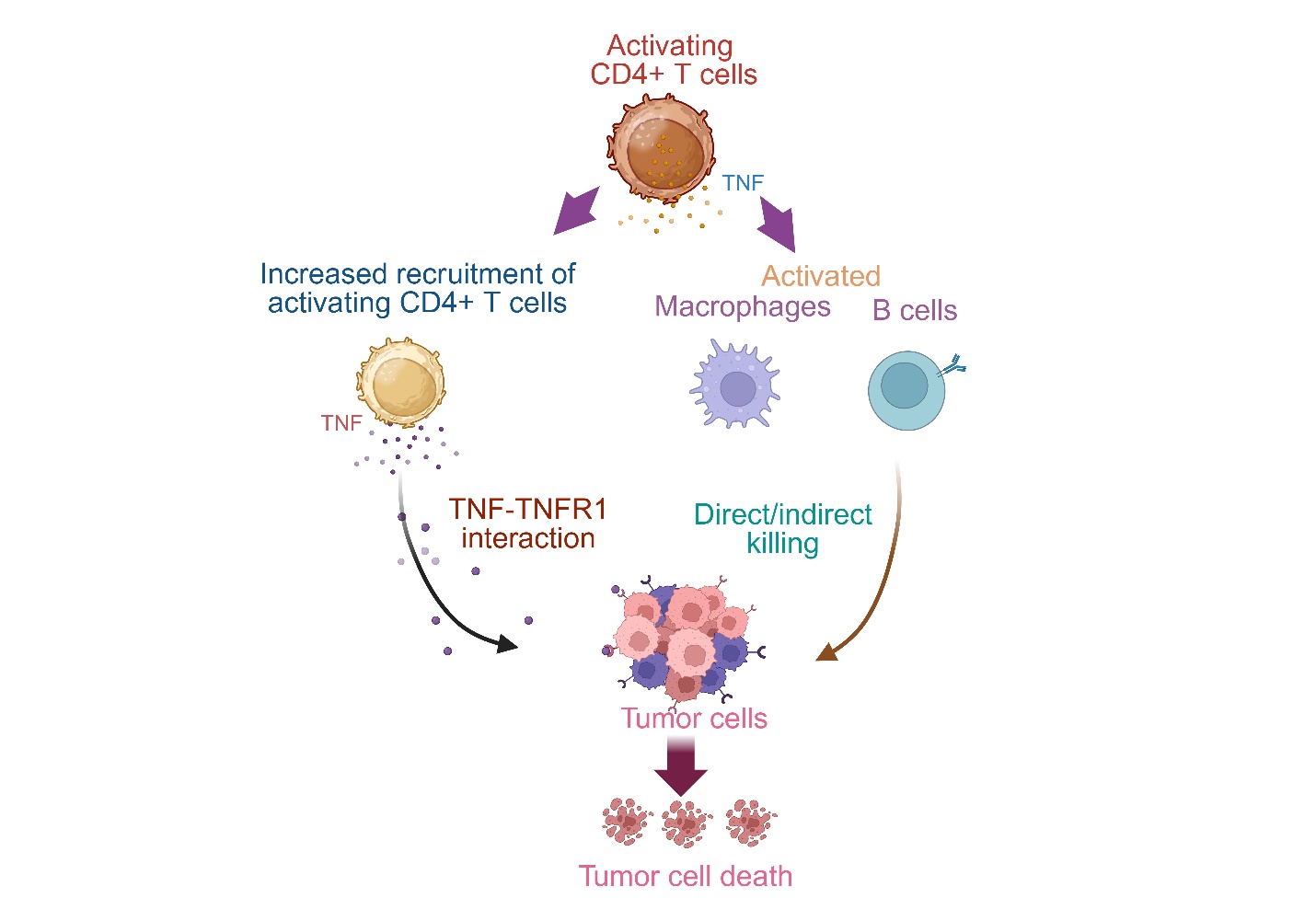
**Supplementary Figure 7.** **Schematic diagram illustrating the molecular mechanism by which T cell-mediated leukemia cell killing**.


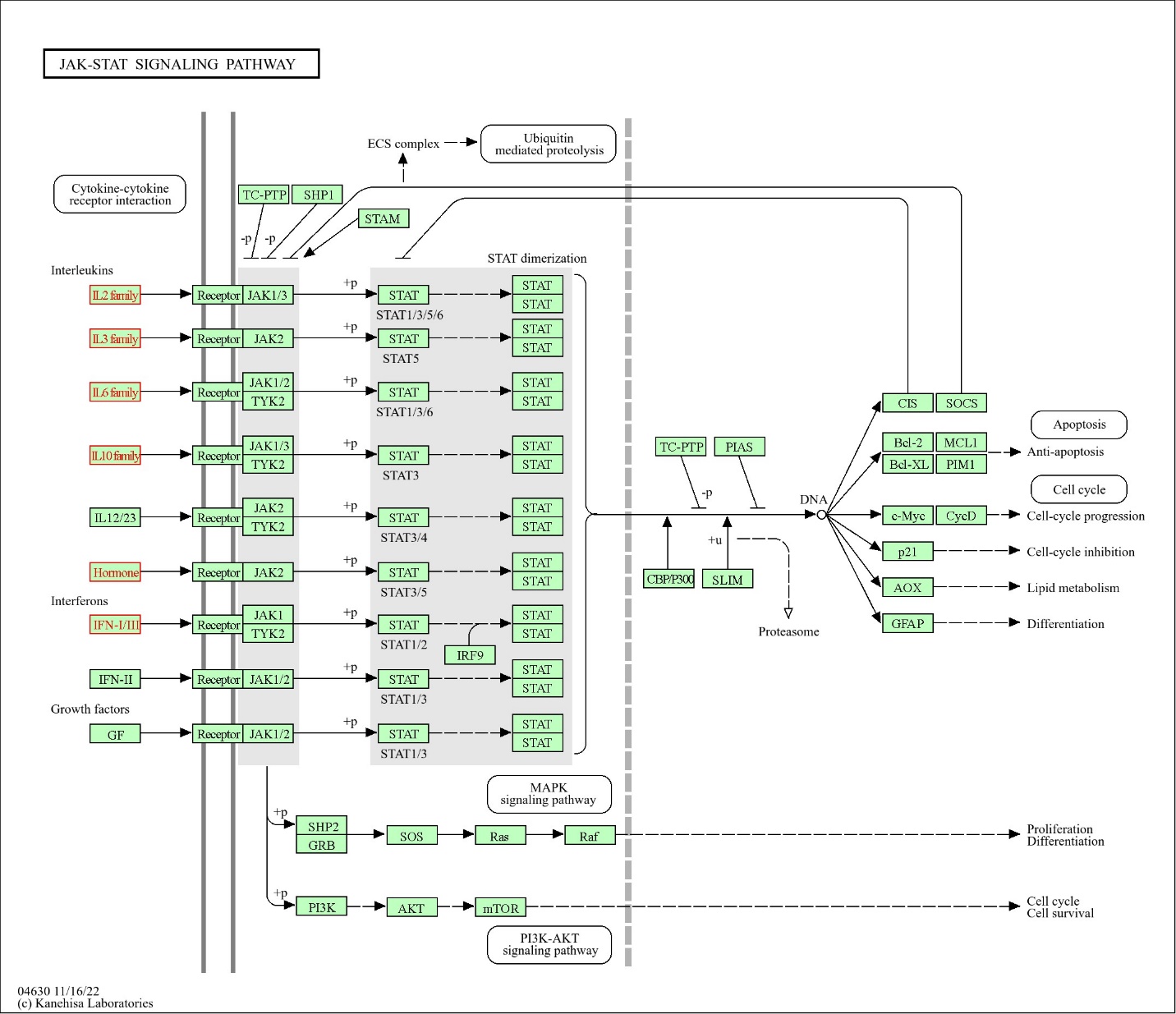


**Supplementary Figure 8. JAK-STAT signaling networks based on differentially expressed cytokines.**


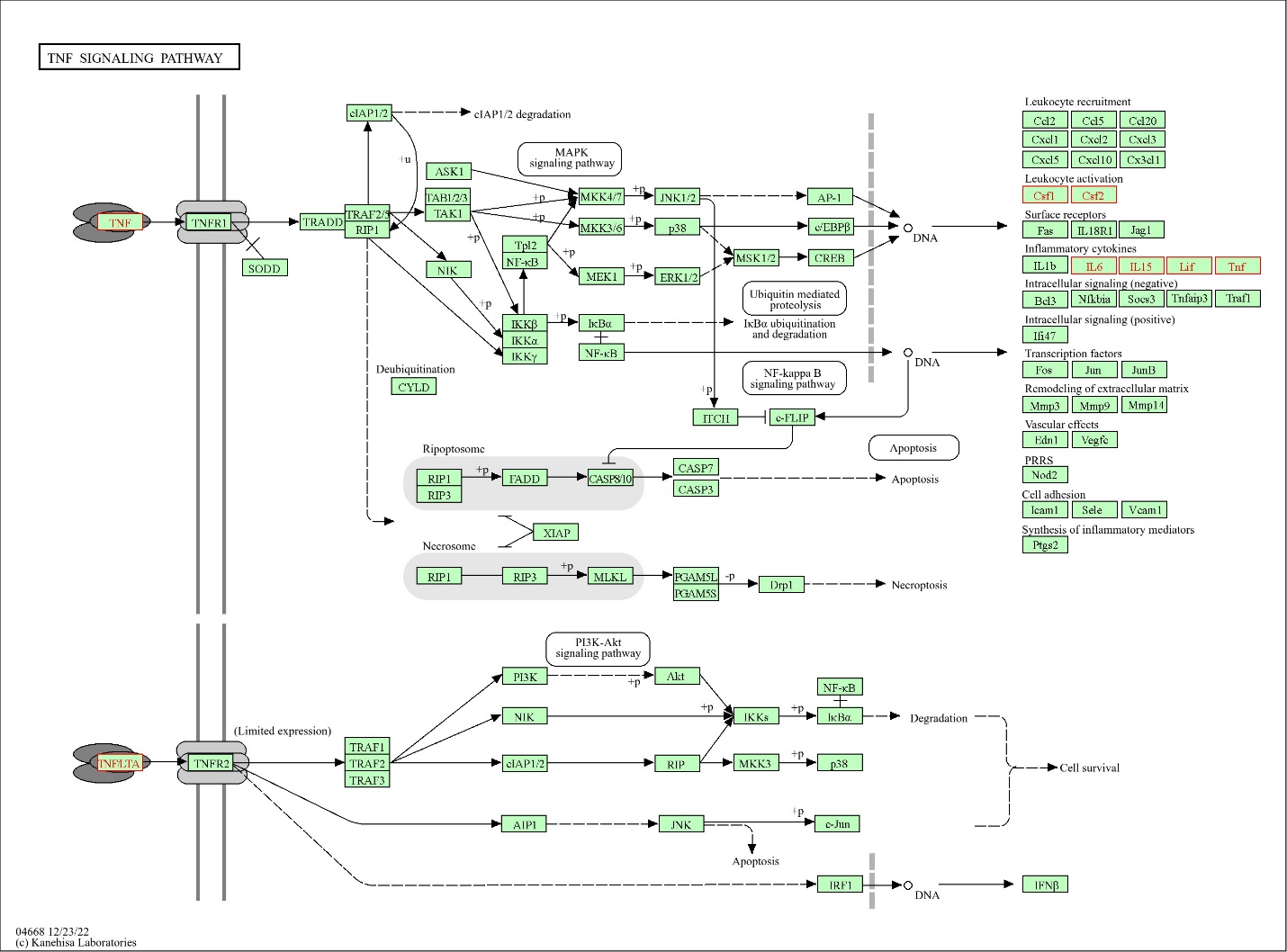
**Supplementary Figure 9. TNF signaling networks based on differentially expressed cytokines.**


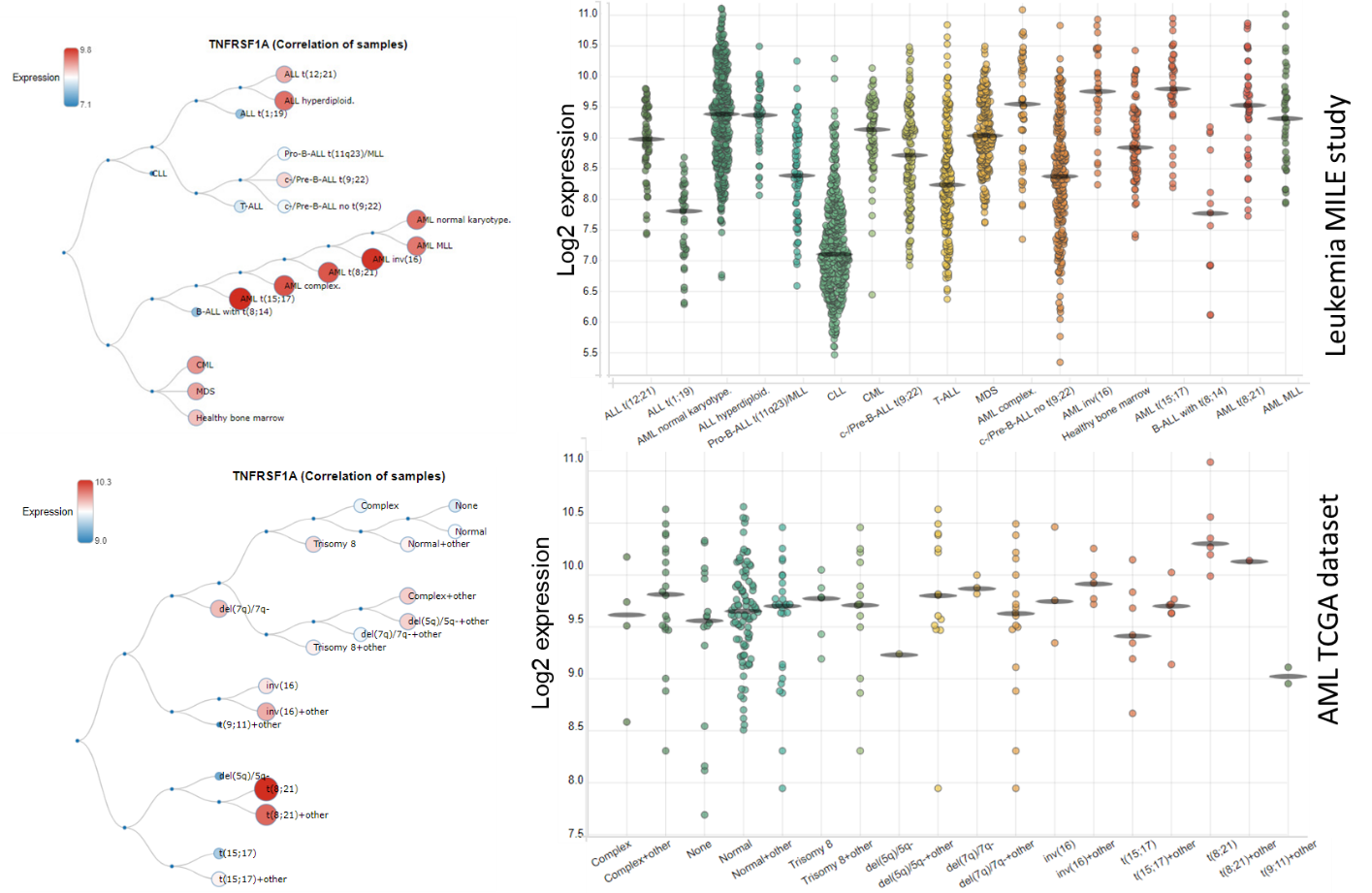


**Supplementary Figure 10. TNFR1 expression in different types of leukemia cells.** The images were generated by online tool Bloodspot eu (https://www.fobinf.com).


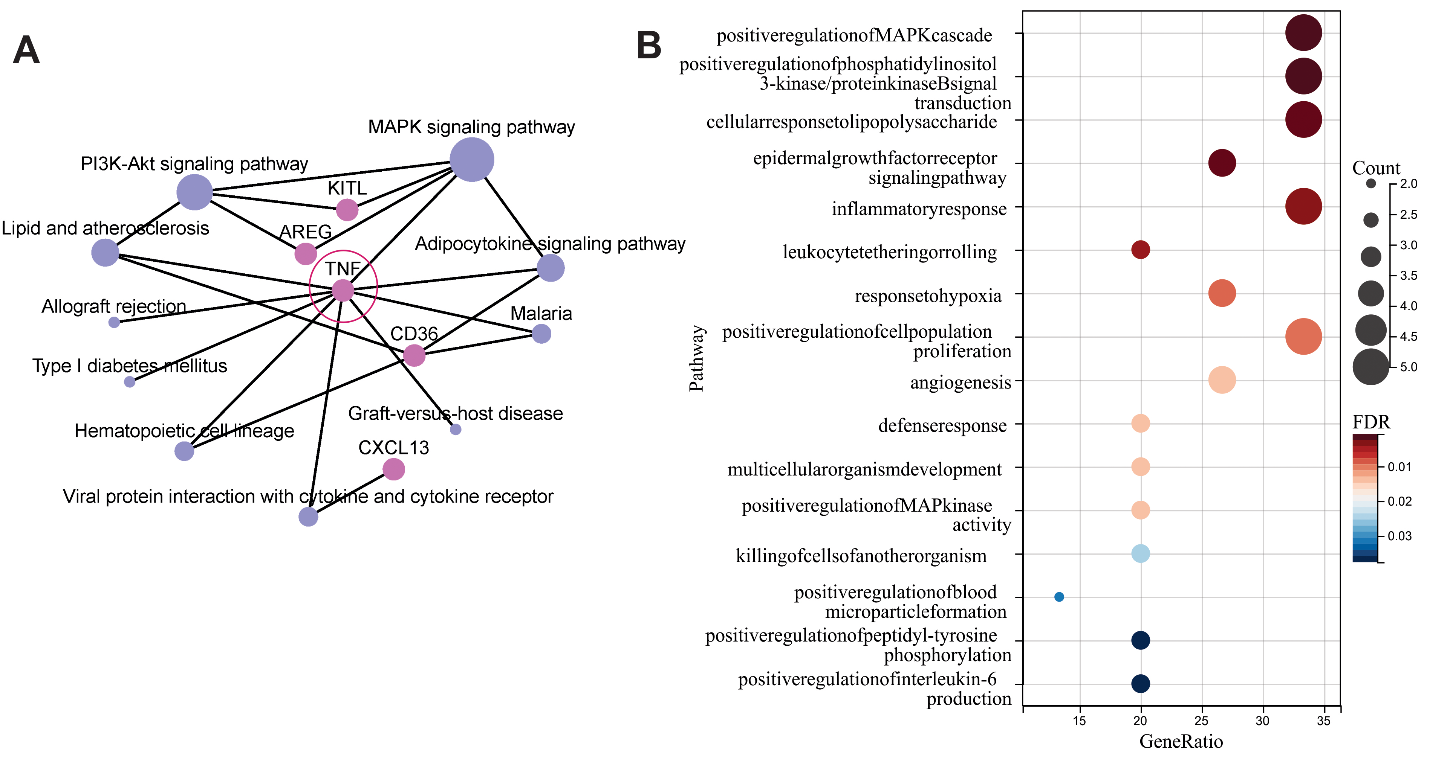
**Supplementary Figure 11.** **Comparison of various plasma cytokines levels between EVs-AE and EV-treated leukemic mice. A** KEGG pathway-based network analysis of cytokines with significantly changed expression (*P* <0.05) in EV-AE vs EV comparison. Nodes identified by the cytokine array are purple, while those defined by KEGG are light blue. Shared nodes across comparisons are highlighted with a red circle (e.g., TNF), with larger circles representing higher enrichment scores. **B** Selected enriched Gene Ontology (GO) terms for cellular and disease-associated functions enriched among up-regulated differentially expressed cytokines. The top 16 terms, ranked by the number of associated DEGs, are presented (FDR <0.05), with dot color indicating the FDR and dot size representing the number of DEGs (map color keys along with dot size ones are shown on the right).

**Supplementary Table 11.** **Analysis of serum factors that are up- or down-regulated in leukemic mice treated with EV only or EV+AE.** Blue text, significantly upregulated serum factors in EV+AE serum compared to EV only serum. Red text, significantly downregulated serum factors in EV+AE mRNA serum compared to EV only serum. Black text, no significant differences between groups. Fold change was calculated as mean EV+AE mRNA fluorescence intensity divided by mean EV only fluorescence intensity for a given factor. *P*-values were calculated by unpaired t test with welch correction. Each pooled sample contains serum from 4 individuals, and the pooled samples were run in quadruplicate. No fold change cut-off was applied, all data are presented.

**Supplementary table 12 Primer sequences used in qPCR**

| Name | (Accession  #/ID) | Primer Sequence (5' to 3') | Primer Sequence (3' to 5') |
| --- | --- | --- | --- |
| Interferon γ | [NM_008337](https://www.ncbi.nlm.nih.gov/nuccore/NM_008337) | CGTTCCTGCTGTGCTTCTCC | TCTTGGAGCTGGAGCTGCTT |
| Interleukin (IL)-6 | [NM_031168](https://www.ncbi.nlm.nih.gov/nuccore/NM_031168) | TACCACTTCACAAGTCGGAGGC | CTGCAAGTGCATCATCGTTGTTC |
| Interleukin (IL)-10 | [NM_010548](https://www.ncbi.nlm.nih.gov/nuccore/NM_010548) | CGGGAAGACAATAACTGCACCC | CGGTTAGCAGTATGTTGTCCAGC |
| TLR-3 | [NM_126166.5](https://www.ncbi.nlm.nih.gov/entrez/viewer.fcgi?db=nucleotide&id=1269612361) | AGCGAGTTTCACTTTCAGGCT | TCTTTTGGTGCGCGATTGTG |
| TLR-7 | [NM_001290755](https://www.ncbi.nlm.nih.gov/nuccore/NM_001290755), | GTGATGCTGTGTGGTTTGTCTGG | CCTTTGTGTGCTCCTGGACCTA |
| TLR- 8 | [NM_133212.3](https://www.ncbi.nlm.nih.gov/entrez/viewer.fcgi?db=nucleotide&id=924181557) | AGTTGGATGTTAAGAGAGAAACAAACG | ATGGCACTGGTTCCAGAGGA |
| ACTIN | [NM_007393.5](https://www.ncbi.nlm.nih.gov/entrez/viewer.fcgi?db=nucleotide&id=930945786) | AGTGTGACGTTGACATCCGT | GCAGCTCAGTAACAGTCCGC |
| AML1/ETO | NM_001024637/NM_004349 | AGCCATGAAGAACCAGG | AGGCTGTAGGAGAATGG |
